# Uniformly Dispersed Fe Clusters on Nitrogen-Doped Carbon Aerogel as a High-Performance Cathode Catalyst for Li-O_2_ Batteries

**DOI:** 10.3390/nano16171055

**Published:** 2026-08-25

**Authors:** Hang Yu, Wenjin Song, Runxin Huang, Jiale Liu, Yanshuo Du, Di Lu, Xianxian Shi, Yufang Chen

**Affiliations:** Department of Materials Science and Engineering, College of Aerospace Science and Engineering, National University of Defense Technology, Changsha 410073, China; yh17891024576@163.com (H.Y.); swj113809@163.com (W.S.); dj2418@126.com (R.H.); liujiale25@nudt.edu.cn (J.L.); dys24@nudt.edu.cn (Y.D.)

**Keywords:** lithium-oxygen batteries, carbon aerogel, iron clusters, bifunctional catalyst, oxygen reduction reaction, oxygen evolution reaction, cycling stability, hierarchical porous structure, non-precious metal catalyst

## Abstract

Lithium-oxygen (Li-O_2_) batteries are a compelling next-generation energy storage candidate owing to their ultrahigh theoretical specific energy, but their practical deployment is critically limited by sluggish cathodic oxygen reduction/evolution kinetics, severe polarization, and poor cyclability. Here, we design a composite catalyst consisting of ultrasmall iron clusters uniformly anchored on a three-dimensional nitrogen-doped carbon aerogel (Fe@NC). The material is synthesized via bidirectional freeze-drying followed by high-temperature reduction carbonization using chitosan, cellulose nanocrystals, and zinc acetate; sublimation of zinc during pyrolysis effectively suppresses iron aggregation, yielding highly dispersed Fe0 clusters of ~10 nm while preserving the aerogel’s hierarchical porous architecture rich in pyridinic and pyrrolic N species. Electrochemical tests show that Fe@NC delivers a deep-discharge specific capacity of 18,000 mAh/g, substantially outperforming pristine carbon aerogel and commercial Ketjen black, and maintains stable cycling over 280 cycles at 500 mAh/g. Microscopic and spectroscopic analyses confirm that Fe@NC promotes uniform, fine-particle Li_2_O_2_ deposition without pore blockage and enables its complete reversible decomposition upon charging, effectively mitigating electrode passivation. This work demonstrates that the synergistic combination of carbon aerogel mass-transport benefits and iron cluster catalytic activity provides a viable, scalable route to high-performance Li-O_2_ battery cathodes.

## 1. Introduction

Lithium-oxygen (Li-O_2_) batteries are regarded as a compelling candidate for next-generation energy storage due to their ultrahigh theoretical specific energy (≈3500 Wh·kg^−1^) [1]. However, their practical application is critically hindered by sluggish cathodic oxygen reduction/evolution reaction (ORR/OER) kinetics, severe polarization, and poor cycling stability [2,3]. In addition, the insulating discharge product Li_2_O_2_ tends to accumulate randomly within the cathode, causing electrode passivation, blockage of active sites, and electrolyte decomposition, which further accelerates capacity degradation [4]. Therefore, designing cathode catalysts that combine efficient bifunctional catalytic activity, facilitate reversible Li_2_O_2_ formation and decomposition, and maintain structural stability remains a core research focus [5,6,7,8,9].

Three-dimensional porous carbon aerogels are considered ideal cathode supports owing to their interconnected conductive networks, hierarchical pore structures, and high specific surface areas [10,11,12,13]. They enable the construction of efficient triphase reaction interfaces, promote mass transport, and provide abundant anchoring sites for active components, outperforming conventional carbon materials such as graphene and carbon nanotubes [14,15,16]. However, pristine carbon aerogels lack sufficient intrinsic catalytic activity and thus require integration with effective catalysts.

Transition-metal-based materials have emerged as promising alternatives to noble metals due to their tunable electronic structures and favorable catalytic compatibility [17,18,19,20]. Among them, iron-based catalysts are particularly attractive because of their abundance, environmental friendliness, and suitable band structures for both ORR and OER [21,22,23,24]. Nevertheless, catalytic performance is highly structure-dependent: single-atom iron (Fe SAs) offers maximum atom utilization but typically suffers from low loading, poor stability, and a tendency to migrate and agglomerate [25,26,27]; large iron nanoparticles (>20 nm) provide better stability but suffer from insufficient exposure of active sites and low atom efficiency due to severe aggregation [28,29]; in contrast, ultrasmall iron clusters (~10 nm) achieve a superior balance between atom utilization, density of unsaturated coordination sites, and stability.

Despite these advantages, achieving uniform dispersion of ultrasmall iron clusters on carbon aerogels remains a significant challenge. To address this, we adopted a bidirectional freeze-drying combined with high-temperature carbonization strategy, using zinc acetate as a pore-forming agent and spacer, whose sublimation during pyrolysis effectively suppresses iron aggregation. This approach successfully yields a nitrogen-doped carbon aerogel loaded with highly dispersed ~10 nm Fe^0^ clusters (Fe@NC), while preserving the original hierarchical porous architecture. This design synergistically combines the mass-transport benefits of the carbon aerogel with the excellent catalytic activity of the iron clusters. Electrochemical tests demonstrate that Fe@NC delivers a specific capacity as high as 18,000 mAh/g and maintains stable cycling over 280 cycles, significantly outperforming pristine carbon aerogel and commercial carbon materials. This work provides a viable technical pathway for developing high-performance, non-precious-metal-based cathodes for lithium-oxygen batteries.

## 2. Materials and Methods

### 2.1. Materials

Chitosan (deacetylation degree ≥ 90%, biochemical reagent grade) was purchased from Solarbio Science & Technology Co., Ltd. (Beijing, China). TEMPO-oxidized cellulose nanocrystals (CNC, freeze-dried powder) were supplied by Colorcom New Materials Co., Ltd. (Hangzhou, China). Deionized water (resistivity ≥ 18.2 MΩ·cm) was produced by a Milli-Q Integral 10 water purification system (Merck Millipore, Darmstadt, Germany) and adopted in all experimental procedures. Iron(II) acetate tetrahydrate (≥98%, ACS reagent grade) was obtained from Macklin Biochemical Co., Ltd. (Shanghai, China). Zinc acetate dihydrate (≥98.5%, ACS reagent grade) was purchased from Aladdin Reagent Co., Ltd. (Shanghai, China). Glacial acetic acid (AR, ≥99.5%) was supplied by Sinopharm Chemical Reagent Co., Ltd. (Shanghai, China). Carbon cloth (Model W1S1009) was purchased from Taiwan Carbon Energy Technology Co., Ltd. (Taiwan, China). Hydrophobic carbon paper (Model TGPH060T) was obtained from Toray Industries, Inc. (Tokyo, Japan).

### 2.2. Preparation of Nitrogen-Doped Carbon Aerogel Loaded with Iron Clusters

**Figure 1:**
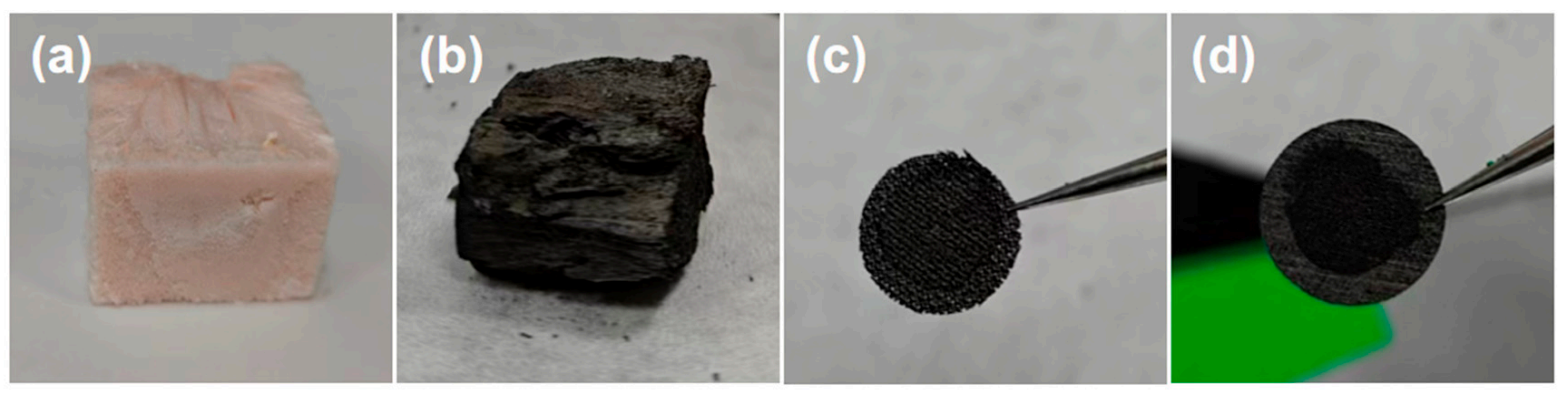
Optical photographs of the experimental process. (**a**) Precursor; (**b**) Carbon aerogel; (**c**) Carbon cloth substrate; (**d**) Carbon paper substrate.

First, 0.3934 g of ferrous acetate was added to 10 mL of deionized water and stirred to obtain a ferrous acetate solution. Separately, 0.191 g of zinc acetate was added to 10 mL of deionized water and stirred to prepare a zinc acetate solution. In a separate container, 1 g of chitosan, 0.2 g of cellulose nanocrystals, and 3 mL of glacial acetic acid were added to 200 mL of deionized water and stirred. Then, 1 mL of the ferrous acetate solution and 1 mL of the zinc acetate solution were introduced. After further stirring until uniform, the mixture was poured into a mold and solidified using a bidirectional freezing method (Figure S1). Finally, freeze-drying for 36 h to obtain the Fe@NC aerogel precursor, as shown in Figure 1a. The precursor was placed in a tube furnace and heated slowly to 850 °C under a 10% H_2_/Ar atmosphere, then held at this temperature for 150 min, yielding the iron-cluster-loaded nitrogen-doped carbon aerogel (Figure 1b).

Prepare a slurry with a ratio of carbon aerogel: conductive carbon black (Super C): PVDF = 8:1:1, transfer it to a φ12 carbon cloth or carbon paper using a pipette, apply 1–2 drops per piece, and dry in vacuum at 85 °C to obtain the positive electrode, with a loading amount of about 0.3–0.5 mg (Figure S2), as shown in Figure 1c,d.

### 2.3. Electrochemical Performance Tests and Material Characterization

The cathode was assembled into a cell with a Whatman glass fiber separator. The electrolyte was 1 M LiTFSI in TEGDME. A lithium foil served as the anode. After assembly, the cell was placed in a sealed Li-O_2_ battery vessel and purged with oxygen. Electrochemical tests were then performed using a LAND battery test system.

The carbon aerogel was characterized by SEM, TEM, and XPS to analyze its microstructure. The cathode electrodes after cycling were examined by SEM and XRD to observe Li_2_O_2_ deposition and decomposition. Detailed information on the testing instruments is provided in the Supporting Information.

## 3. Results and Discussion

### 3.1. Microstructural Characterisation of Fe@nc

**Figure 2:**
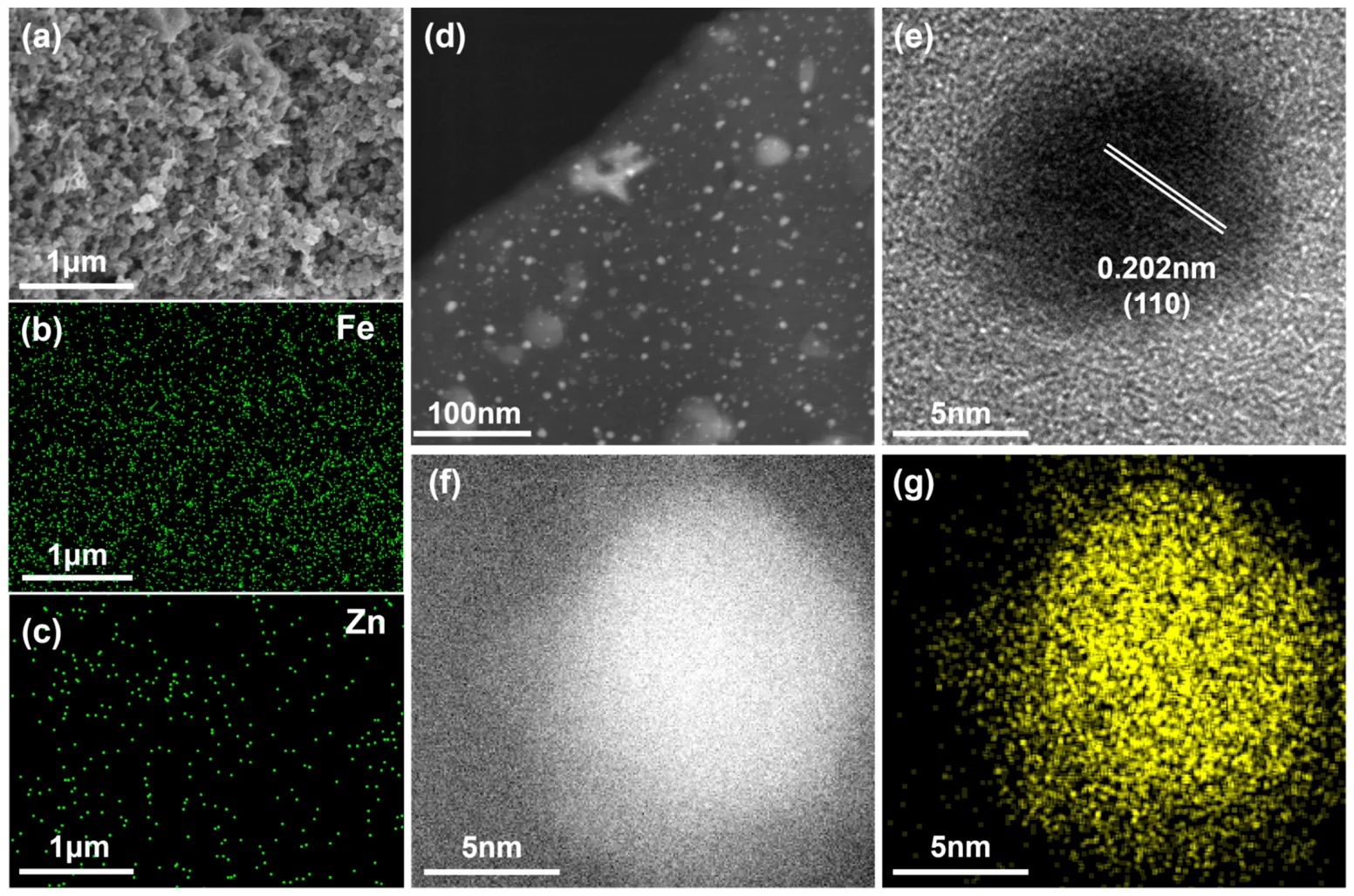
Morphology and cluster distribution of Fe@NC. (**a**–**c**) SEM images and corresponding EDS mappings; (**d**,**e**) HR-TEM images and corresponding EDS mappings; (**f**,**g**) AC-TEM images and corresponding EDS mappings.

The morphology and structure of the as-prepared Fe@NC were characterized by SEM and EDS, as shown in Figure 2a–c. SEM images reveal that the prepared Fe@NC possesses an excellent pore structure, which provides sufficient space for the deposition of the discharge product Li_2_O_2_. EDS mapping shows that Fe is uniformly distributed on the surface of the carbon aerogel, while Zn is nearly absent. To further investigate the growth and distribution of Fe clusters, HR-TEM was performed on Fe@NC (Figure 2d,e). The results indicate that the Fe clusters are uniformly dispersed without obvious aggregation. This is attributed to the addition of Zn, which suppresses Fe agglomeration, leading to uniform clusters with appropriate sizes.

The measured interplanar spacing is 0.202 nm, corresponding to the (110) plane of the BCC phase. Subsequently, AC-TEM and EDS analyses (Figure 2f,g) reveal that the Fe clusters are approximately 10 nm in size and exhibit a regular spherical shape.

In summary, this method successfully loads iron clusters with a size of about 10 nm onto the nitrogen-doped carbon aerogel.

**Figure 3:**
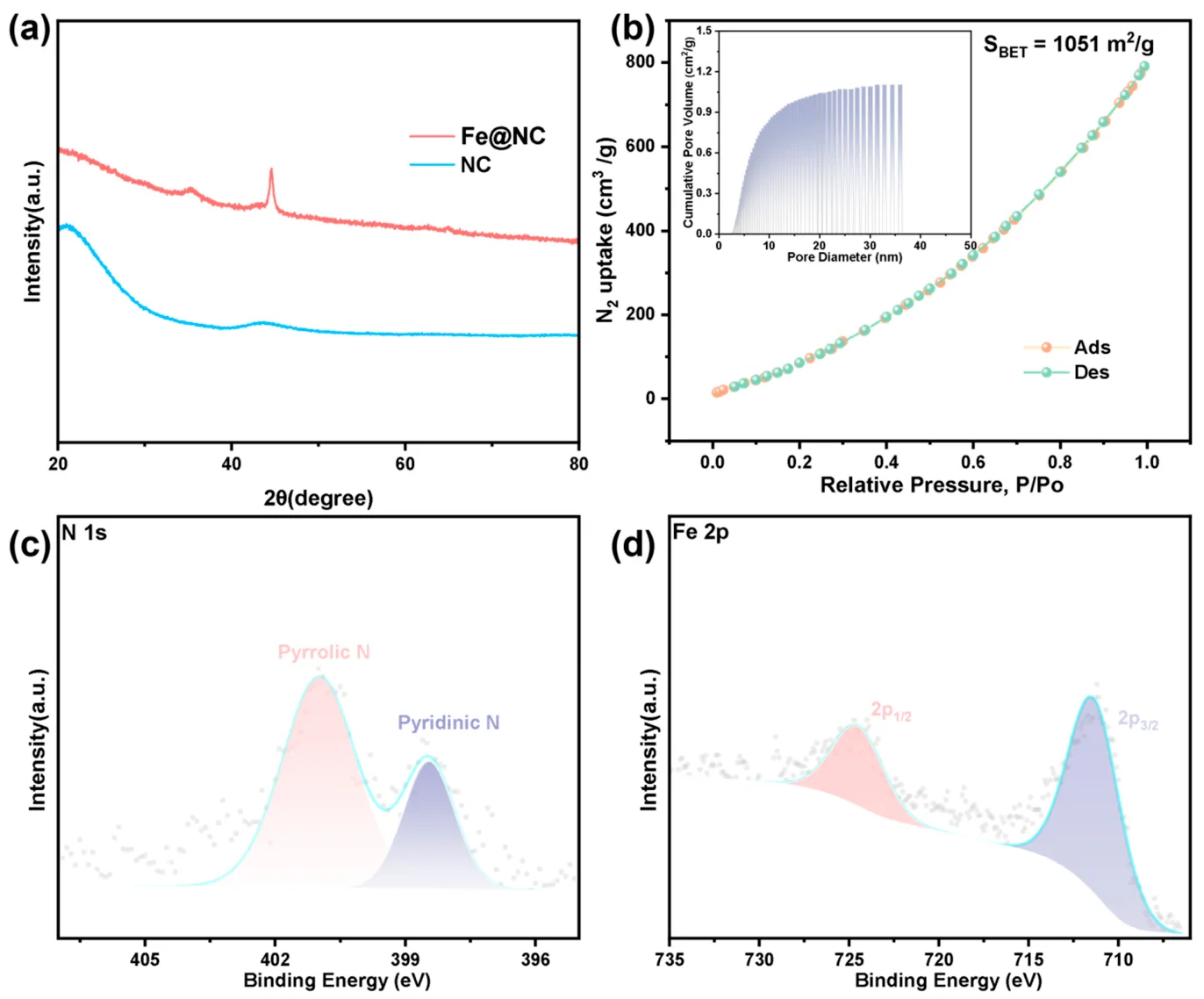
Structural characterization of Fe@NC. (**a**) XRD patterns; (**b**) BET; (**c**,**d**) XPS spectra.

To further investigate the structure and composition of Fe@NC, XRD, FT-IR, and XPS analyses were conducted, as shown in Figure 3.

The XRD pattern (Figure 3a) reveals that Fe@NC exhibits an additional distinct peak at 2θ ≈ 44.7° compared with NC, which corresponds to the (110) plane of α-Fe. A weak peak at 65.1° is also observed, assigned to the (200) plane. The FT-IR spectra (Figure S4) show no significant differences between Fe@NC and NC, indicating that the loading of iron clusters does not alter the functional groups or structure of the carbon aerogel. Both samples exhibit identical characteristic bands originating from oxygen- and nitrogen-containing functional groups derived from chitosan carbonization. The dual peaks at 2200–2340 cm^−1^ are assigned to C≡N dopant groups and ambient CO_2_ interference. Peaks at 1600–1800 cm^−1^ arise from conjugated C=C, C=N. The broad absorption at 1200–1500 cm^−1^ originates from C-N single bonds. To investigate the pore structure of NC, BET measurements were performed in Figure 3b. The results reveal a specific surface area (S_BET_) of 1051 m^2^/g and pore sizes ranging from 5 to 35 nm. This hierarchical structure, comprising both mesopores and micropores, facilitates Li_2_O_2_ deposition. In contrast, commercial carbon materials like Ketjen Black (KB) are predominantly microporous, which can easily block pore channels and aggravate polarization.

The XPS spectra are presented in Figure 3c,d and Figure S5. The C 1s spectrum (Figure S5) shows that carbon in Fe@NC mainly exists as C-C and C-N, with the C-N originating from chitosan in the precursor. The N 1s spectrum (Figure 3c) reveals that nitrogen is present as pyrrolic N and pyridinic N. These nitrogen species may introduce asymmetric charge distributions and defect sites, serving as catalytic active centers and also as anchoring points for iron cluster deposition. The Fe 2p spectrum (Figure 3d) indicates that Fe exists in its metallic state, confirming that the reducing atmosphere effectively reduces Fe^2+^ to the desired Fe^0^ clusters during preparation. The Zn 2p spectrum (Figure S5) shows that zinc is nearly absent in Fe@NC, because zinc metal sublimes at high temperature, leaving only metallic iron clusters.

### 3.2. Catalytic and Electrochemical Characterization of Fe@nc

**Figure 4:**
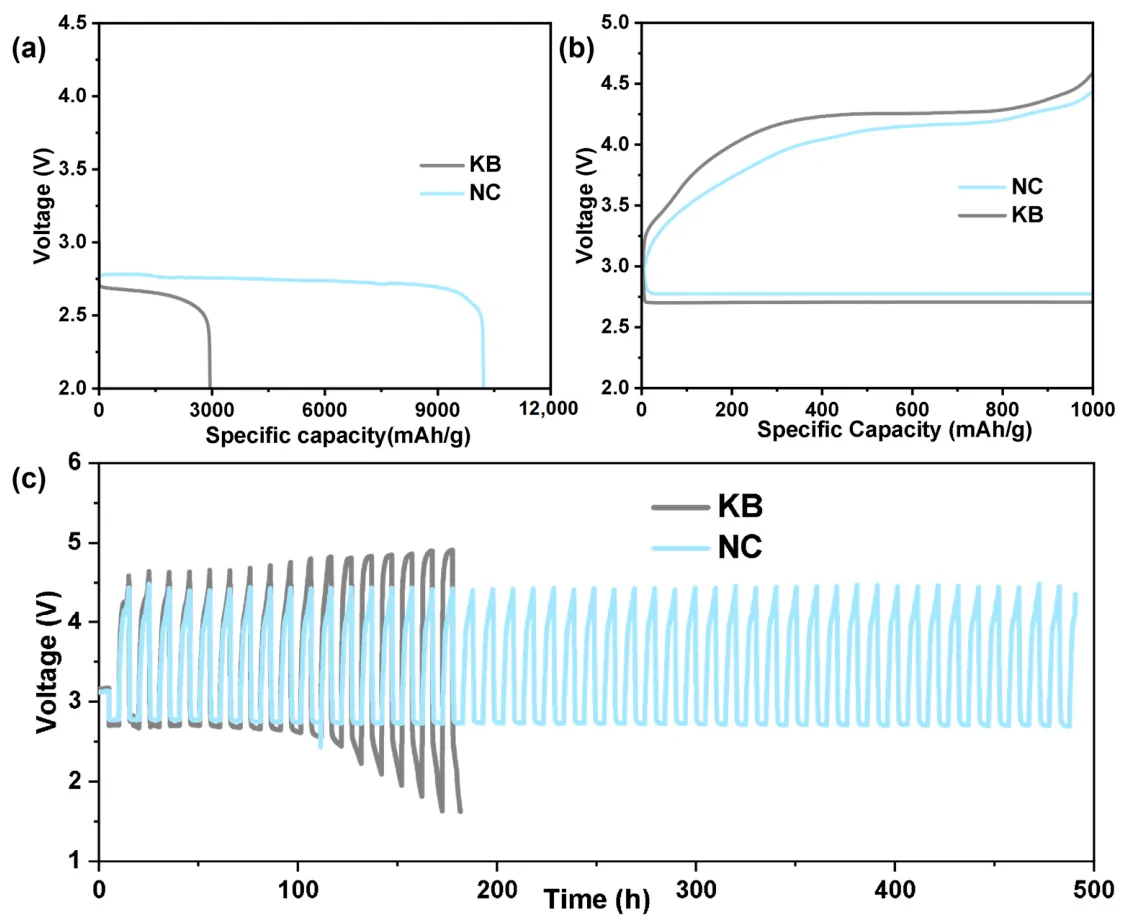
Catalytic and electrochemical performance of NC. (**a**) Deep discharge; (**b**) overpotential; (**c**) cycling performance.

The carbon aerogel support and commercial carbon material KB were compared, as shown in Figure 4. The deep discharge tests (Figure 4a) reveal that NC exhibits a significantly higher discharge capacity than KB. A comparison of their overpotentials (Figure 4b) also shows that NC has a substantially lower overpotential than KB. These results indicate that NC possesses better catalytic activity and more Li_2_O_2_ deposition space than KB, which is attributed to its excellent pore structure and abundant N-doping sites.

Cycling test results are presented in Figure 4c. KB shows a sharp increase in overpotential after only 10 cycles. In contrast, NC maintains the voltage within 2.5–4.5 V for 40 cycles with no obvious change in overpotential. This further confirms that NC exhibits superior catalytic performance compared with KB. Considering the performance variations caused by N doping, we employed N-doped carbon nanotubes (N-CNTs), obtained by pyrolyzing aminated carbon nanotubes, as a catalyst. Deep discharge (Figure S6) and cycling tests (Figure S7) were conducted. The results showed that the performance of CNTs was substantially inferior to that of NC. This indicates that employing a hierarchically porous aerogel as a catalyst offers marked advantages.

**Figure 5:**
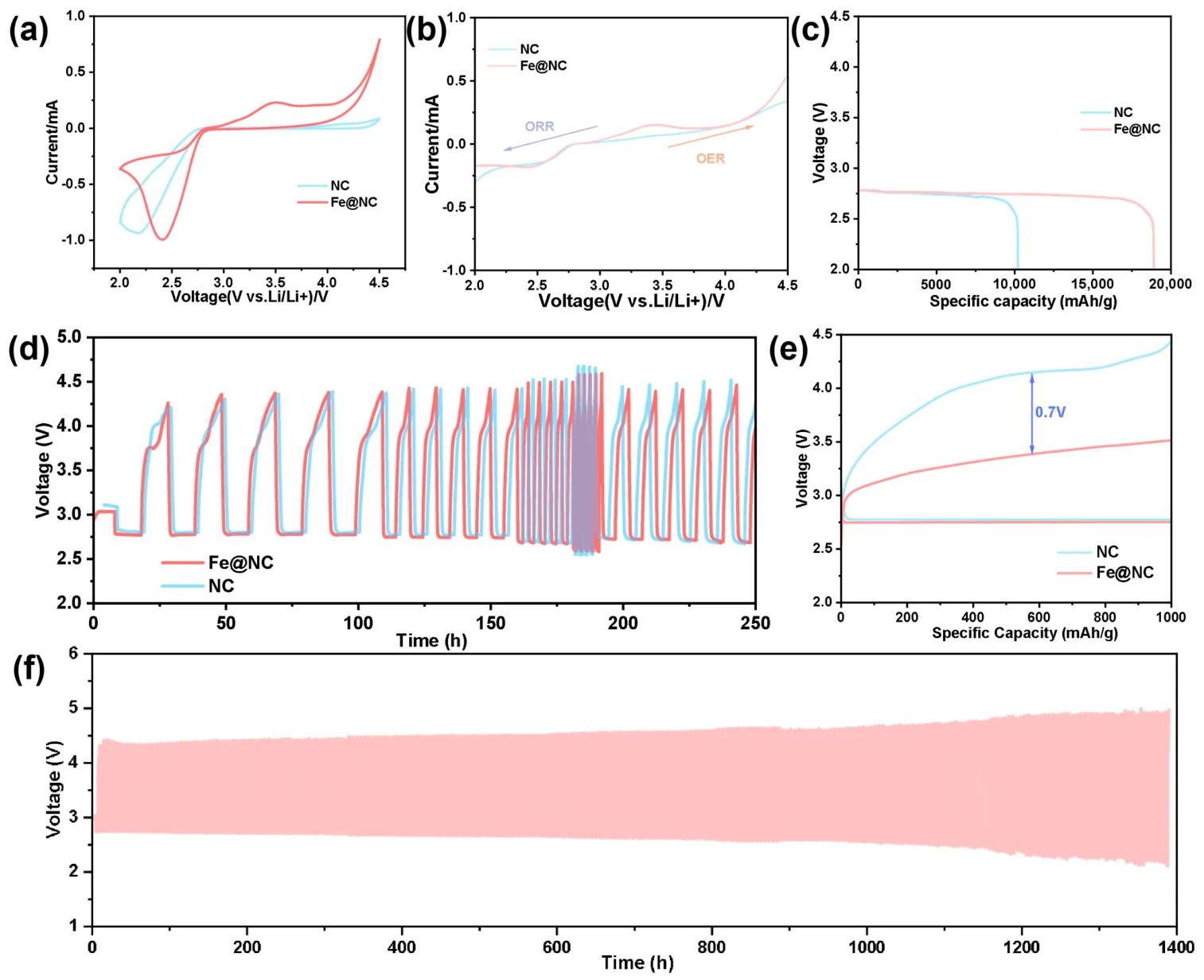
Catalytic and electrochemical performance of Fe@NC. (**a**) CV; (**b**) LSV; (**c**) deep discharge; (**d**) rate capability; (**e**) overpotential; (**f**) cycling performance.

To further investigate the effect of iron cluster loading on the bifunctional ORR/OER catalytic performance for Li-O_2_ batteries, electrochemical tests were conducted, as shown in Figure 5.

The CV results are presented in Figure 5a. The potential range of 3.0–2.0 V corresponds to the ORR stage. In this stage, Fe@NC exhibits a reduction peak potential closer to 3.0 V than NC, indicating a lower ORR overpotential. The absolute reduction peak current of Fe@NC is much larger than that of NC, suggesting superior ORR kinetics and more facile O2 reduction. Thus, the loading of Fe clusters significantly improves the ORR performance. For the OER stage (3.0–4.5 V), Fe@NC shows a much higher oxidation current than NC, with a distinct oxidation peak around 3.5 V, corresponding to Li_2_O_2_ decomposition. In contrast, the current of NC remains nearly unchanged in this region, indicating that the bare carbon support cannot effectively catalyze the OER and fails to achieve reversible Li_2_O_2_ decomposition. For Fe@NC, the current rises rapidly in the 4.0–4.5 V range, indicating continuous OER progression and thus excellent OER catalytic activity. The LSV results are presented in Figure 5b. Compared with NC, Fe@NC exhibits a more favorable onset potential and a larger reduction current density, indicating accelerated OER kinetics. In the potential range of 3.2–4.5 V, Fe@NC shows an earlier oxidation onset and significantly enhanced oxidation current across the entire potential window, suggesting that the Fe catalytic sites greatly lower the decomposition barrier of Li_2_O_2_.

Rate capability tests are displayed in Figure 5d. At a fixed capacity of 1000 mAh/g, Fe@NC exhibits lower overpotentials than NC (Figure 5e), indicating that Fe cluster loading significantly reduces the charge overpotential. This is consistent with the CV results, especially the differences observed in the OER region. Deep discharge tests were then performed, as shown in Figure 5c. After discharging to 2.0 V, NC delivers a specific capacity of approximately 10,000 mAh/g, much higher than that of KB. This is attributed to its excellent pore structure and abundant N-doping sites, which accommodate more Li_2_O_2_ deposition. Fe@NC achieves a specific capacity of about 18,000 mAh/g, a marked improvement over NC, indicating that Fe cluster loading optimizes Li_2_O_2_ deposition and thus enhances capacity. Cycling performance is presented in Figure 5f. The tests were performed at a constant capacity of 500 mAh/g with a galvanostatic current of 200 mA/g. Fe@NC maintains its voltage within 2–5 V even after 280 cycles, demonstrating excellent cycling stability. Combined with the CV results, this can be attributed to the superior OER performance of Fe@NC compared with NC, which enables thorough Li_2_O_2_ decomposition.

### 3.3. Morphology and Reversibility of the Reaction Products of Fe@NC

**Figure 6:**
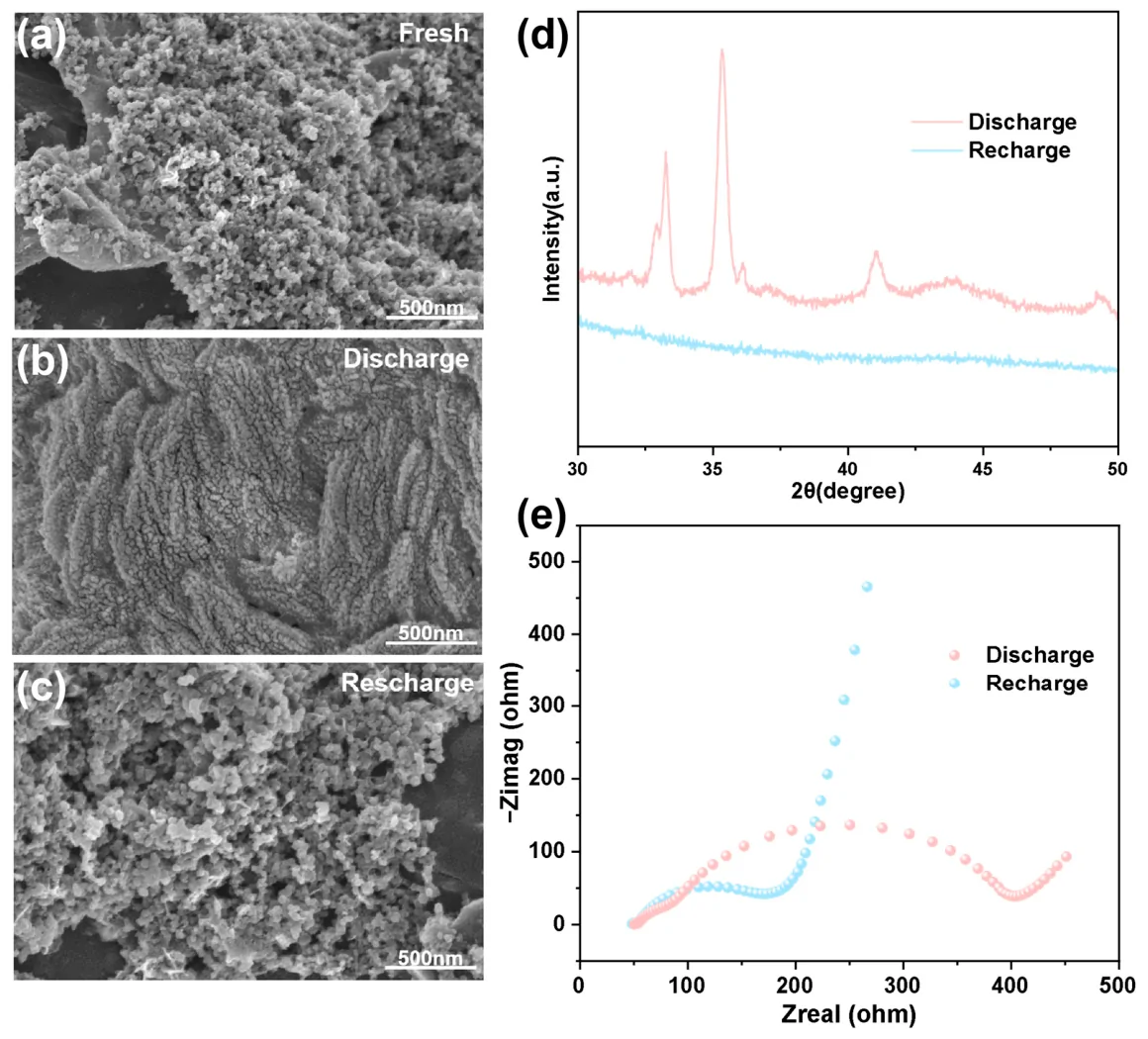
Effect of Fe@NC on Li_2_O_2_ deposition and reversible decomposition. (**a**–**c**) SEM; (**d**) XRD; (**e**) EIS.

To investigate the influence of Fe@NC on Li_2_O_2_ deposition and its reversible decomposition, the cathode electrodes after the first discharge and after recharge were examined. The results are presented in Figure 6.

SEM was performed on the electrodes after discharge and after recharge to observe the morphology of the deposited Li_2_O_2_, as shown in Figure 6a–c. Figure 6a shows the SEM image of the pristine cathode. Figure 6b displays the SEM image after deep discharge. On the Fe@NC catalyst, Li_2_O_2_ appears as small particles uniformly attached to the carbon skeleton. This growth mode maximizes the advantage of the aerogel’s high specific surface area and does not block the pores. This explains the exceptionally high specific capacity of Fe@NC observed in the deep discharge tests. Figure 6c shows the SEM image after recharge. The Fe@NC catalyst recovers its original carbon framework, and Li_2_O_2_ is almost completely decomposed. The SEM results indicate that the Fe@NC catalyst promotes the reversible decomposition of Li_2_O_2_.

XRD analysis was also performed on both electrodes, as shown in Figure 6d. After deep discharge, distinct Li_2_O_2_ peaks appear at 2θ ≈ 32.5° and 35.5°, corresponding to the (100) and (101) planes, respectively. After recharge, these peaks disappear, indicating complete Li_2_O_2_ decomposition.

EIS measurements were conducted on the batteries after discharge and after recharge, as shown in Figure 6e. The impedance is higher after discharge, which is attributed to the insulating nature of Li_2_O_2_. The Li_2_O_2_ coverage on the cathode increases polarization. After charging, the impedance returns to the normal state, confirming Li_2_O_2_ decomposition.

Table 1 lists the electrochemical performance of several materials from analogous systems, including cobalt clusters, single atoms, iron-based alloys, and Fe–N co-doped carbon materials. It is evident that the cycling performance of our work is comparable to that of the other reported systems.

## 4. Conclusions

In this work, we prepared Fe@NC by bidirectional freeze-drying and carbonization of chitosan and cellulose nanocrystals with zinc acetate as a spacer. Zinc sublimation prevents Fe agglomeration, yielding ~10 nm Fe^0^ clusters, while the carbon aerogel retains abundant pyridinic/pyrrolic N sites and a 3D hierarchical porous structure for efficient triphase interfaces. Fe@NC outperforms NC and KB in ORR/OER activity, reduces polarization, delivers 18,000 mAh/g capacity, and shows stable cycling. Microscopy confirms that Li_2_O_2_ deposits as fine particles and decomposes reversibly upon charging, with impedance recovery and no passivation. The synergy between carbon aerogel and Fe clusters enhances mass transfer and lowers kinetic barriers, improving reversibility. With abundant raw materials and simple processing, this work addresses poor kinetics and rapid decay, offering a feasible strategy for non-precious metal cluster-based porous carbon cathodes for Li-O_2_ batteries.

## Figures and Tables

**Table 1:** Comparison of electrochemical properties of LOB of some similar materials.

Materials	Specific Capacity	Cycle Count (n)	References
Co@NC	500 mAh/g	230	[30]
N-HP-Co SACs	1000 mAh/g	260	[31]
Nb-Fe-NCNT	1000 mAh/g	290	[32]
N-wdC-MS (Fe)	0.5 mAh/cm^2^	190	[33]
This work	500 mAh/g	280	--

## Data Availability

The original contributions presented in this study are included in the article/Supplementary Material. Further inquiries can be directed to the corresponding authors.

## Supplementary Materials

The following supporting information can be downloaded at: https://www.mdpi.com/article/10.3390/nano16171055/s1, Figure S1: Liquid-nitrogen-based bidirectional freezing mold; Figure S2: Optical image of the coated carbon paper and the corresponding mass loading; Figure S3: TEM images and corresponding EDS mappings; Figure S4: FT-IR spectra; Figure S5: XPS spectra: Zn 2p; Figure S6: Galvanostatic deep discharge/charge profiles of CNT; Figure S7: Cycling performance of CNT.

## Abbreviations

The following abbreviations are used in this manuscript:

NC	Nitrogen-doped carbon aerogel
Fe@NC	Nitrogen-doped carbon aerogel-supported iron clusters
CNT	Carbon nanotube
KB	Commercial Ketjen black
