# Supplementary material for: Uniformly Dispersed Fe Clusters on Nitrogen-Doped Carbon Aerogel as a High-Performance Cathode Catalyst for Li-O2 Batteries
Source: Nanomaterials (Basel). 2026 Aug 25;16(17):1055. doi: 10.3390/nano16171055 (PMC13567109; doi:10.3390/nano16171055)
Supplement: Supplementary file 1 [file nanomaterials-16-01055-s001.zip › nanomaterials-4495171-supplementary.pdf]

Supporting

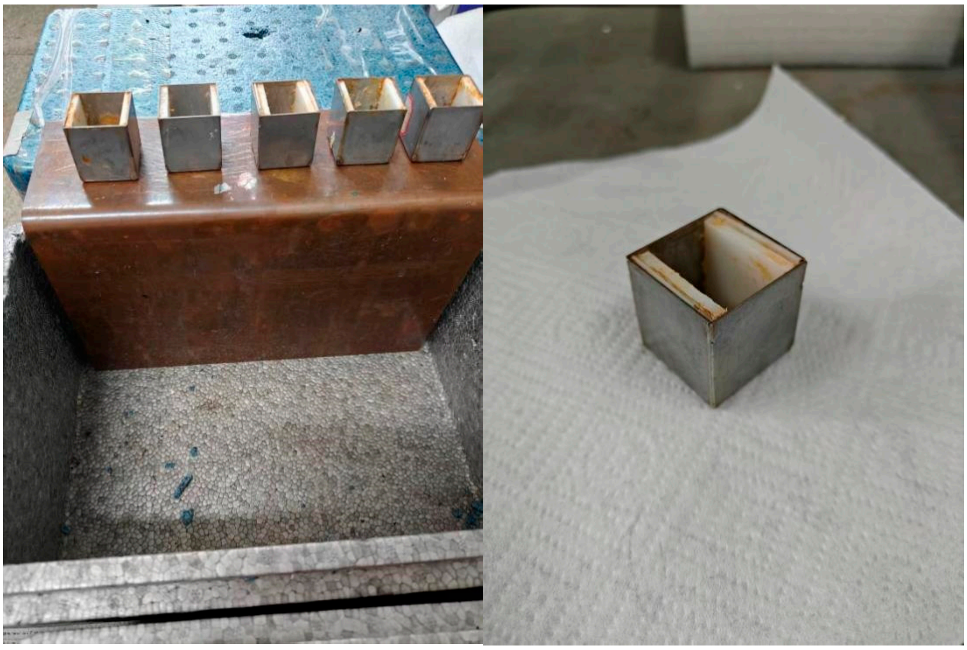

Figure S1 Liquid-nitrogen-based bidirectional freezing mold

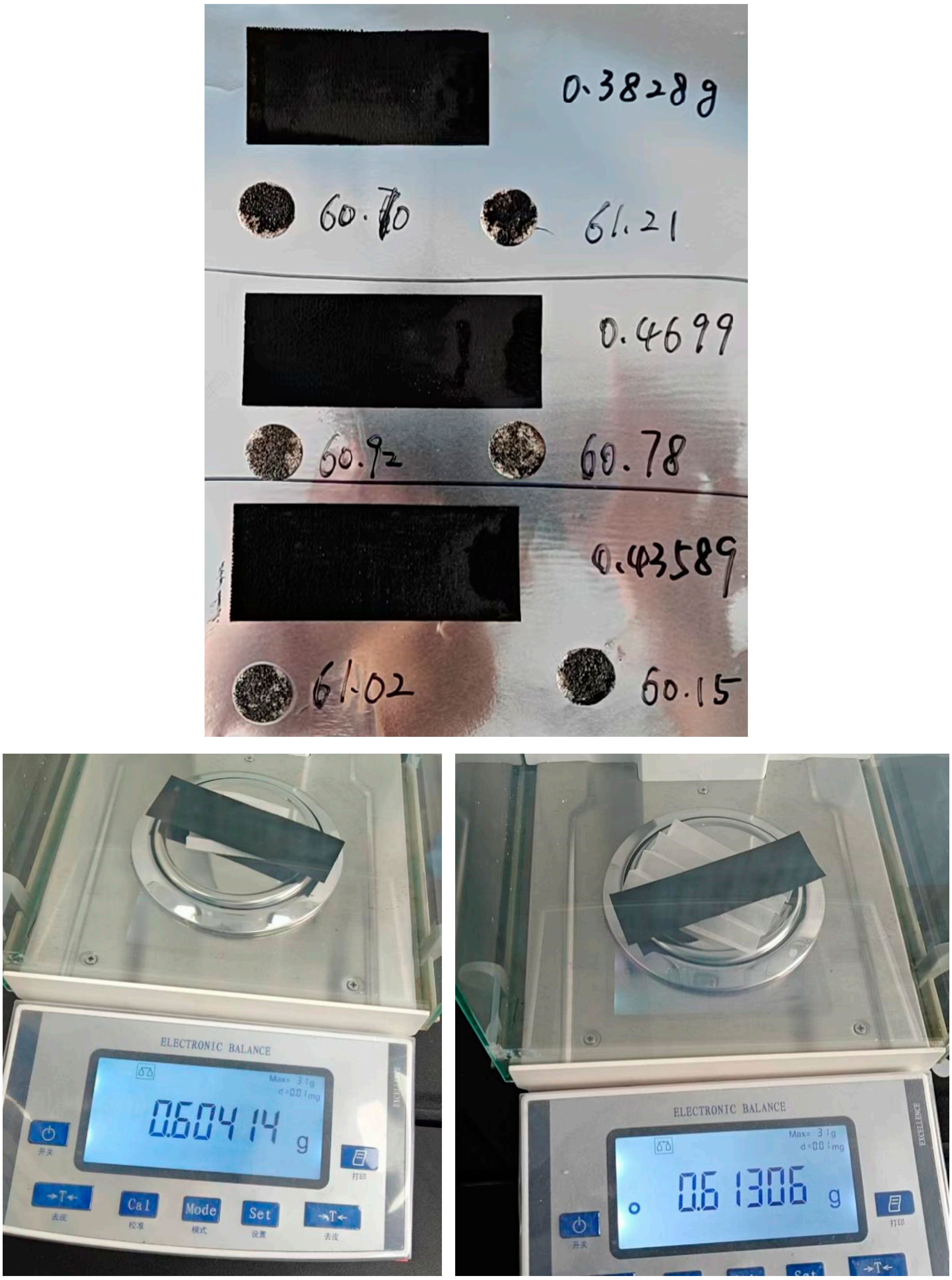

Figure S2 Optical image of the coated carbon paper and the corresponding mass loading

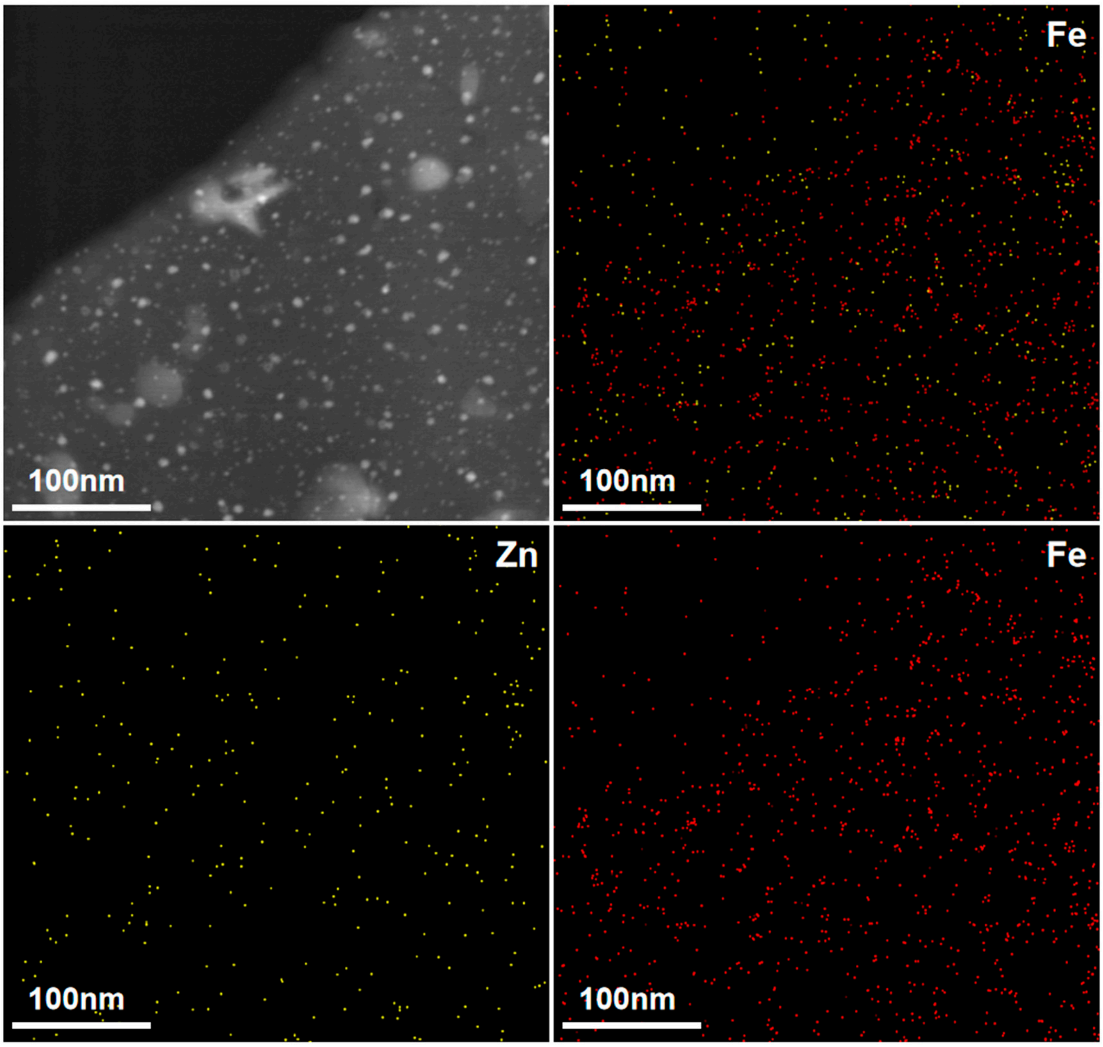

Figure S3 TEM images and corresponding EDS mappings

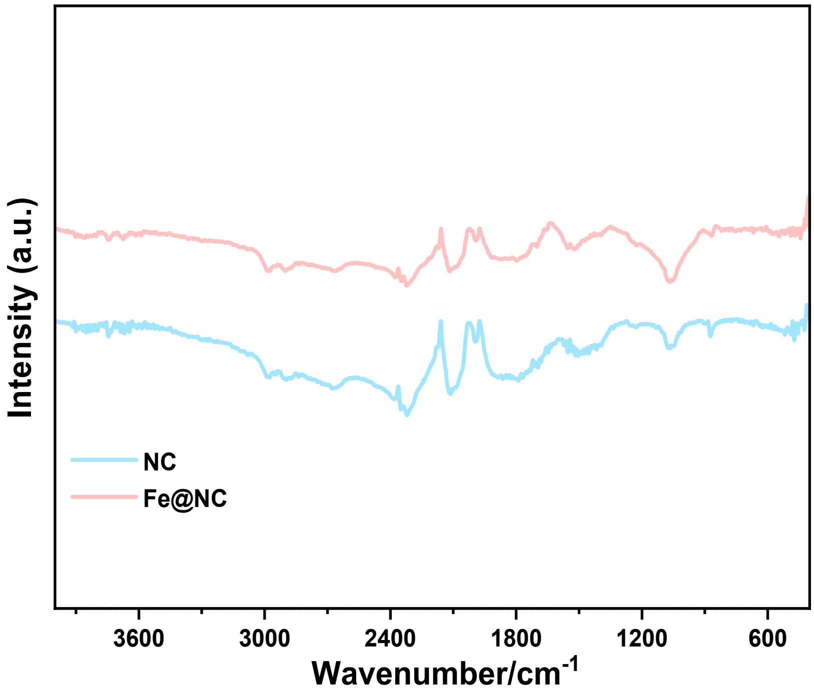

Figure S4 FT-IR spectra.

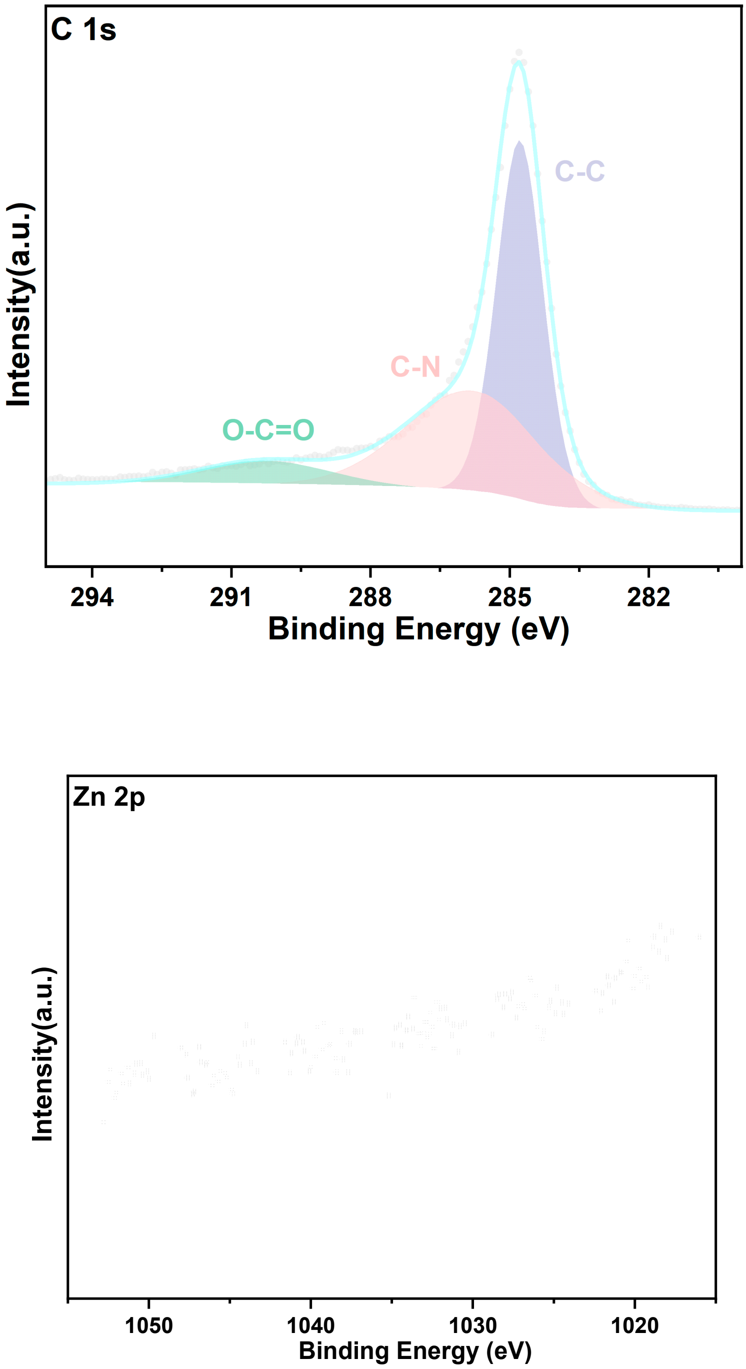

Figure S5 XPS spectra: C、Zn

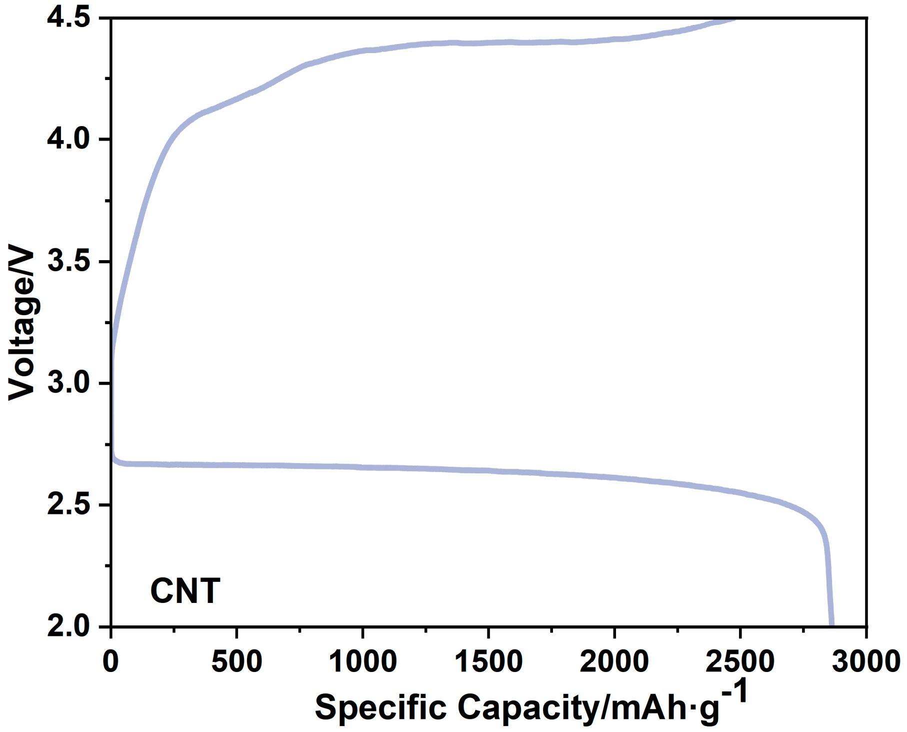

Figure S6 Galvanostatic deep discharge/charge profiles of N-CNTs

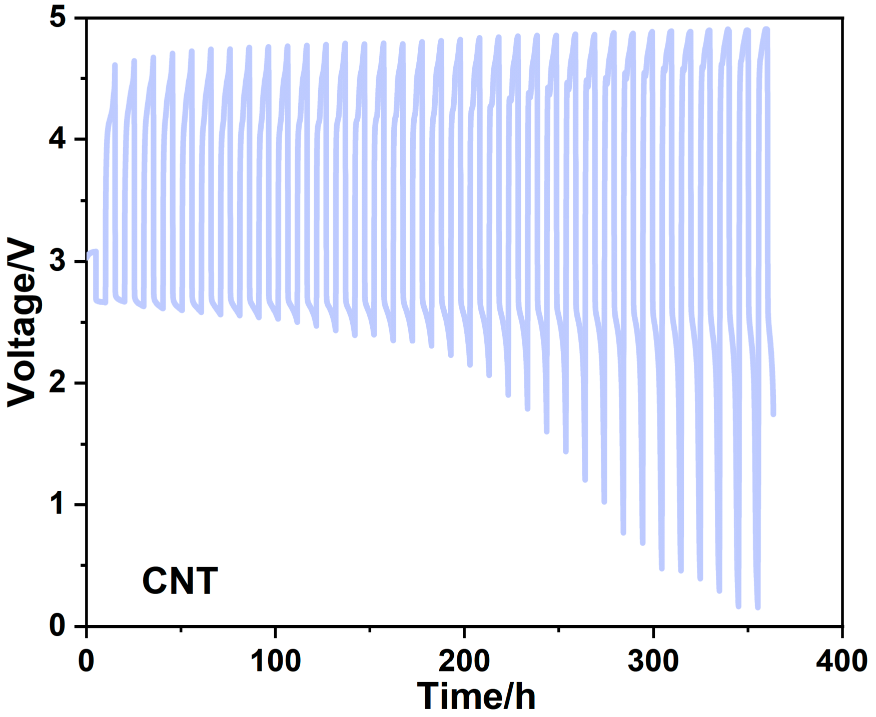

Figure S7 Cycling performance of N-CNTs

Instruments and models used in the experiment: Milli-Q Integral 10 ultrapure water machine, high-temperature tube furnace, vacuum freeze dryer, DZF-6050 vacuum drying oven, Hitachi SU8010 field emission scanning electron microscope (equipped with Oxford X-Max EDS), FEI Tecnai G2 F20 high-resolution transmission electron microscope, Rigaku Ultima IV X-ray diffractometer, Nicolet iS50 Fourier transform infrared spectrometer, Thermo ESCALAB 250Xi X-ray photoelectron spectrometer, LAND CT2001A battery testing system, Gamry electrochemical workstation
